# Investigations of Graphene and Nitrogen-Doped Graphene Enhanced Polycaprolactone 3D Scaffolds for Bone Tissue Engineering

**DOI:** 10.3390/nano11040929

**Published:** 2021-04-06

**Authors:** Weiguang Wang, Jun-Xiang Chen, Yanhao Hou, Paulo Bartolo, Wei-Hung Chiang

**Affiliations:** 1 Department of Mechanical, Aerospace and Civil Engineering, School of Engineering, Faculty of Science and Engineering, The University of Manchester, Manchester M13 9PL, UK; yanhao.hou@manchester.ac.uk (Y.H.); paulojorge.dasilvabartolo@manchester.ac.uk (P.B.); 2 Department of Chemical Engineering, National Taiwan University of Science and Technology, Taipei E2-514, Taiwan; roy0936742266@gmail.com

**Keywords:** additive manufacturing, biomanufacturing, electro-active scaffolds, extrusion process, doping, graphene, polycaprolactone, tissue engineering

## Abstract

Scaffolds play a key role in tissue engineering applications. In the case of bone tissue engineering, scaffolds are expected to provide both sufficient mechanical properties to withstand the physiological loads, and appropriate bioactivity to stimulate cell growth. In order to further enhance cell–cell signaling and cell–material interaction, electro-active scaffolds have been developed based on the use of electrically conductive biomaterials or blending electrically conductive fillers to non-conductive biomaterials. Graphene has been widely used as functioning filler for the fabrication of electro-active bone tissue engineering scaffolds, due to its high electrical conductivity and potential to enhance both mechanical and biological properties. Nitrogen-doped graphene, a unique form of graphene-derived nanomaterials, presents significantly higher electrical conductivity than pristine graphene, and better surface hydrophilicity while maintaining a similar mechanical property. This paper investigates the synthesis and use of high-performance nitrogen-doped graphene as a functional filler of poly(*ɛ*-caprolactone) (PCL) scaffolds enabling to develop the next generation of electro-active scaffolds. Compared to PCL scaffolds and PCL/graphene scaffolds, these novel scaffolds present improved in vitro biological performance.

## 1. Introduction

Biomanufacturing is a relatively new research domain focusing on the use of additive manufacturing technologies, biomaterials, cells, and biomolecular signals to produce constructs for tissue engineering applications. These tissue constructs (scaffolds) play an important role for cell attachment, proliferation, and differentiation, ultimately leading to new tissue formation. Bone scaffolds must be biocompatible; biodegradable, with a degradation rate that ideally should match tissue regeneration; presenting adequate mechanical properties to support physiological loads once implanted in the body; high porosity allowing good permeability and tissue ingrowth; and must be bioactive to stimulate and accelerate the regeneration process [1,2,3].

In order to mimic the native bone structure and properties, bioceramics and bioglasses have been widely used due to their biocompatibility, bioactivity, and high mechanical strength [4]. However, they may present limited biodegradability, are brittle, and difficult to process [5]. To overcome these limitations, biocompatible and biodegradable polymers are the most commonly used materials in the field. Usually, polymeric materials are blended with bioceramics, bioglasses or stimuli-responsive biomaterials such as electrical conductive carbon nanomaterials or magnetic nanoparticles, to improve physical and biological properties [6,7,8,9,10].

These materials are usually processed using additive manufacturing, which gradually replaces conventional manufacturing technologies such as solvent casting, salt leaching, and electrospinning, as it allows the fabrication of scaffolds with high accuracy and reproducibility [11,12]. 

A wide range of functional fillers have been investigated for the fabrication of high-performance bone tissue engineering scaffolds. The incorporation of magnetic nanoparticles such as iron oxide nanoparticles have been used to create magnetic stimuli-responsive scaffolds, showing improved hydrophilicity and mechanical properties, as well as the ability to form an apatite layer promoting bone [13,14,15]. However, these scaffolds require the use of an external ultrasound source and there are some concerns related to cytotoxicity and long-term effects related to the bioaccumulation of the magnetic nanoparticles [16]. Carbon nanomaterials have been used to create electro-active scaffolds. A particularly relevant carbon nanomaterial is Graphene (G), which has been explored for a wide range of applications such as sensors [17], lithium-ion batteries [18], dye-sensitized solar cells [19], and capacitors [20], owing to the remarkable electrical, optical, thermal, and mechanical properties as well as high surface area (~2630 m^2^g^–1^) [21]. G present better physical interaction with cells compared with other commonly used carbon nanomaterials (e.g., carbon nanotubes) due to its 2D-like shape (higher ratio of peripheral to central carbon atoms) and superior electrical conductivity under low volume percentage, showing a strong potential for in vitro and in vivo biological applications [22,23,24]. Previous studies proved that additive manufacturing can successfully fabricate electro-active bone tissue engineering scaffolds based on the combination of PCL and G. Results showed that the addition of small concentrations of G can enhance the mechanical properties of PCL scaffolds, and promote in vitro cell proliferation [25,26], differentiation [27], and in vivo bone regeneration [28]. One possible mechanism enhancing osteogenesis through electrical stimulation involves the up-regulation of intercellular calcium concentration through the activation of voltage-gated Ca^2+^ modulating osteogenesis via calmodulin pathways [29]. Moreover, bone is dynamically remodeled by signaling pathways, controlled by cells and the extra cellular matrix, and transmitted through an electrical and chemical synapse. Usually, implanted scaffolds disrupt these natural signaling pathways. However, previous studies show that polymer/G scaffolds, due to their electrical conductivity, are able to preserve signal conduction, contributing to bone formation even without the use of any external electrical stimulation source [28,30]. Studies also demonstrated that the interactions between G and cells mainly depend on the physicochemical and electrical properties of G, such as morphology, number of layers, surface properties, functionalization groups, and the method of synthesis [31,32]. These factors can disturb the mechanism of cell uptake and tissue response, affecting cell viability, reactive oxygen species generation, and gene expression [33,34]. It was also reported that the in vitro and in vivo cytotoxic of G is dependent on shape, dose, and cell–material interaction time [35].

Doping G with heteroatoms such as nitrogen (N) and/or boron (B) is an effective approach to further enhance the performance of G, including its electronic, surface properties, and biological responses [36]. Recently, nitrogen-doped graphene (N-G) with tuneable surface polarities, strong electron withdrawing ability, and negligible environmental and biological hazards [37,38,39,40,41], showed a strong potential for a wide range of applications, including biological sensing, bioimaging, drug delivery, catalysis, and renewable energy generation [42,43,44,45]. N-G appears to be a promising bioactive material with superior sensitivity, selectivity, and applicability for biological and medical applications [46,47], compared with conventional organic fluorescent dyes, toxic semiconductor quantum dots, and expensive noble-metal nanoparticles [46,48]. Moreover, the surface hydrophilicity of graphene can be further modified by nitrogen doping [49]. However, the synthesis of N-G at atmospheric-pressure, its biological properties, and the use of N-G for bone tissue engineering applications has not yet been investigated. Therefore, this paper investigates material synthesis, and physical and chemical properties of G and N-G. Moreover, PCL/G and PCL/N-G blends containing the same concentration of G and N-G were prepared for the fabrication of electro-active bone tissue engineering scaffolds. Surface hydrophilicity and in vitro biological were conducted to investigate the effect of N-G with respect to G.

## 2. Materials and Methods

### 2.1. Material Preparation

PCL was selected as the base material for the electro-active scaffold fabrication due to its biocompatibility, biodegradability, and ease of process by material-extrusion additive manufacturing. Three millimeter PCL pellets (Capa^TM^ 6500, Perstorp, Warrington, UK), with 1.1 g/cm^3^ density and 60 °C melting temperature were used in this research.

G was prepared using liquid-phase exfoliation with graphite as previously reported by our group [50]. In brief, the graphite powder was first mixed with a solvent containing 0.8 mass fraction of N-methyl-2-pyrrolidone (NMP) and 0.2 mass fraction of water, and the graphite concentration was controlled at 5 mg/mL. Then, the mixed solution was sonicated for 6 h in a bath sonicator with a 100 W nominal power and 37 kHz frequency, followed by centrifugation (3000 rpm, 30 min). The colloidal supernatant was further collected by filtration and dried at 50 °C to yield G. 

**Figure 1:**
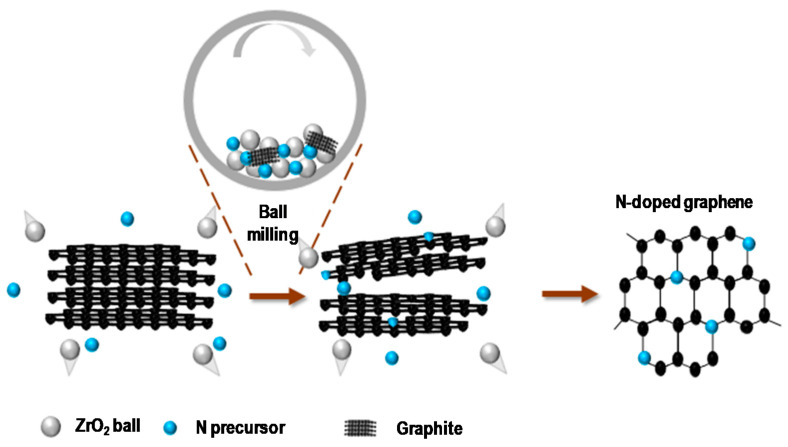
Schematic illustration of the preparation of N-G through ball milling.

N-G was synthesized through ball milling of graphite and melamine as the nitrogen precursor using a planetary ball-mill machine (Emax, Restch, Haan, Germany) (Figure 1). 0.5 g of graphite and 0.25 g of melamine were added into a stainless-steel grinding jar (100 mL) containing 300 ZrO_2_ grinding ball (3 mm). The jar was sealed at ambient conditions followed by installing it in the planetary ball-mill machine. The mixture was then ball milled with 900 rpm for 5 h. After ball milling, the prepared product was washed with 80 °C water several times, followed by filtration with a polytetrafluoroethene (PTFE) membrane with a pore size of 1 μm. The filtered solid sample was then collected and dried at 60 °C to yield the N-G nanosheets. The obtained G and N-G presented similar morphology including 1–3 nm thickness and 400~600 nm surface lateral size. 

### 2.2. Scaffold Fabrication

PCL/G pallets and PCL/N-G pallets were prepared through a physical melt-blending process, and three different concentrations of G and N-G (1, 3, and 5 wt.%) were considered. Briefly, PCL pellets were heated up to 150 °C in a crucible, allowing a molten state, and carbon nanomaterial fillers were added at different designed concentrations. The mixed materials were stirred for 20 min to guarantee a homogenous dispersion. After cooling to room temperature, the obtained materials were cut into small pieces suitable for printing.

A material-extrusion additive manufacturing system (3D Discovery, RegenHU, Villaz-Saint-Pierre, Switerzland) was considered for the fabrication of all scaffolds. To ensure reproducible scaffold manufacturing with a constant filament diameter close to the nozzle diameter (330 μm), the following optimal processing parameters were considered: 92 °C of melting temperature, 270 μm of layer thickness, screw rotation velocity of 8 rpm, and deposition velocity of 13 mm/s. The designed filament distance was 680 μm, and the lay-down pattern was 0°/90° to obtain square shape pores. The overall dimensions of fabricated scaffolds are 32 mm × 32 mm × 3.2 mm.

### 2.3. Characterization of Graphene

#### 2.3.1. Scanning Electron Microscopy, Transmission Electron Microscopy and Atomic Force Microscopy

To study the surface morphology of the raw material before synthesis (graphite), and after synthesis (G and N-G), as well as scaffold morphology, scanning electron microscopy (SEM) observations were performed using a field emission SEM with a 15 kV accelerating voltage (FESEM, 6700F, JEOL, Tokyo, Japan). Transmission electron microscopy (TEM) was utilized to further investigate the morphology and nanostructure of G and N-G, using a field emission TEM (Philips Tecnai F20 G2 FETEM, Thermo-Fisher Scientific, Waltham, MA, USA) with 200 kV accelerating voltage. SEM and TEM samples were prepared by solution dry-casting of the colloidal solution on silicon wafer and carbon-coated copper grids (300 mesh, Ted Pella Inc., Redding, CA, USA) respectively. Furthermore, atomic force microscopy (AFM) was also considered to additionally probe the mechanochemical cracking of a large thickness of graphite into thin grapheme-like nanostructures. The P-100 AFM system (Ardic instruments, Taipei, Taiwan) was used with a tapping mode and the samples were prepared by ethanol solution and spin coating on mica substrates.

#### 2.3.2. X-ray Photoelectron and Raman Spectroscopy 

X-ray photoelectron (XPS) was considered to investigate the chemical content and configuration of N-G. Furthermore, the atomic-scale structural information of G and the density of defects induced by N doping in the N-G were obtained from micro Raman measurements. Thin films of G and N-G were formed on silicon wafers by drop coating and drying in ambient conditions for 24 h for both XPS and Raman spectroscopy measurements. XPS was carried out using a VG ESCA Scientific Theta Probe (UK). The pass energy and take off angle was 50 eV and 53° respectively. In addition, the beam size was 400 μm for a Al Kα (1486.6 eV) radiation as the excitation source. Raman measurements were performed with a JASCO 5100 spectrometer (λ = 533 nm) (Japan) under room temperature. To avoid the effect of laser heating, the laser power was maintained at 0.2 mW.

#### 2.3.3. Electrical Conductivity Measurement

Thin films of G and N-G were prepared to determine the electrical conductivity of G and N-G. Five milligrams of the sample was added into 20 mL deionized water, and the solution was dispersed by using a high energy homogenizer (disperser T-10, IKA, Königswinter, Germany) at 75 W for 10 min. Vacuum filtration of the as-prepared dispersions was then performed using a polyvinylidene difluoride (PVDF) filter with 0.2 μm pore size and 47 mm diameter (Pall Corporation, Port Washington, NY, USA). Finally, the films were dried at ambient conditions for 24 h, and the sheet resistance was measured by a commercial electrical four-point meter (MCP-T610, Loresta-GP, Mitsubishi Instrument Inc., Tokyo, Japan) with a PSP type probe (1.5 mm inter-pin distance). The areas of the films were divided into 10 measured positions to determine the average values.

### 2.4. Characterization of Scaffolds

#### 2.4.1. Thermogravimetric Analysis

Thermogravimetric analysis (TGA) was considered to evaluate the actual carbon nanomaterial concentration present in the scaffold after fabrication. Thermal Analysis Q500 analyzer (TA Instrument, New Castle, DE, USA) was used, and tests were conducted in air atmosphere with 60 mL/min airflow rate. The experimental temperature ranged from room temperature up to 590 °C with a 5 °C/min increasing rate. Scaffolds were cut into 2 g samples and the weight change was recorded by TA Universal Analysis 2000 software (TA Instrument, USA).

#### 2.4.2. Surface Hydrophilicity Characterization

To evaluate the surface hydrophilicity of all printed scaffolds, static apparent water-in-air contact angle (WCA) analysis was considered using a KSV CAM 200 system (KSV Instruments, Espoo, Finland). The sessile drop method was used, and deionized water droplets of ~1 mL were dropped on the surface of the scaffold using a micrometric liquid dispenser (Hamilton, Reno, NV, USA). The shapes of the droplets were recorded with a high-speed framing camera (DMK 21F04 FireWire monochrome camera, Imaging Source, Bremen, Germany), and analyzed by using the Attension Theta software (Biolin Scientific, Gothenburg, Sweden) to obtain contact angle values.

#### 2.4.3. In Vitro Biological Characterization

Scaffolds were cut into small blocks (11 mm × 11 mm × 3.2 mm) to fit in 24-well culture plates. All samples were sterilized with 70% ethanol for 1 h, rinsed with phosphate buffered saline (PBS) (Sigma-Aldrich, Gillingham, Dorset, UK), and air-dried for 24 h in an incubator at 37 °C prior to the cell seeding. Human adipose-derived stem cells (hADSCs) (Invitrogen, Waltham, MA, USA) (passages 4–6) were used to evaluate the cytotoxicity and cell–material interaction of all fabricated scaffolds. 5 × 10^4^ hADSCs (counted by Cellometer Auto 1000 Bright Field Cell Counter (Nexcelom Bioscience, Lawrence, MA, USA)) were seeded to each scaffold, and cultured in 0.8 mL MesenPRO RS™ basal medium (Thermo Fisher Scientific, Waltham, MA, USA) under standard conditions (37 °C, 5% CO_2_ and 95% humidity).

Evaluations were conducted at 1, 3, 7, and 14 days after cell seeding, using the Alamar Blue assay, to quantitatively monitor the cytotoxicity of the scaffolds. At each test point, scaffolds were first transferred to a new well plate, and then 0.8 mL of medium containing 0.001% Resazurin sodium salt (Sigma-Aldrich, Gillingham, Dorset, UK) was added to each well. The incubation was conducted for 4 h under standard conditions. Then 150 μL of medium from each well was transferred into a 96-well plate and the fluorescence intensity was measured by a Multi-Detection Microplate Reader Synergy HT (BioTec, Minneapolis, MN, USA) (540 nm excitation wavelength and 590 nm emission wavelength). 

## 3. Results and Discussion

### 3.1. Graphene Characterisation

**Figure 2:**
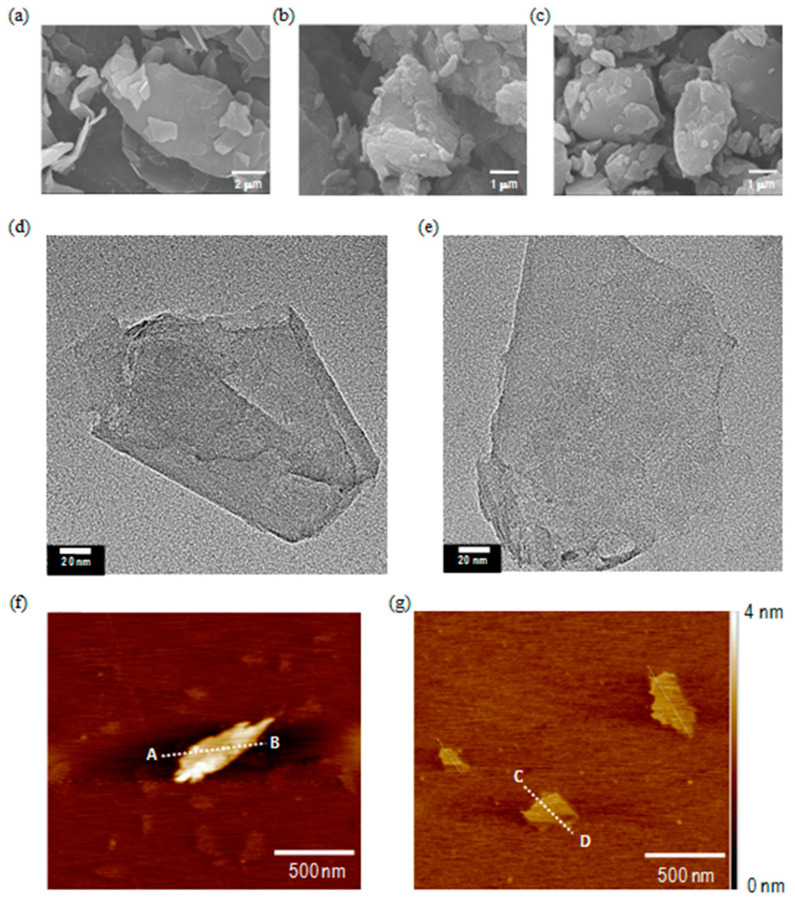
SEM images of (**a**) graphite, (**b**) G, and (**c**) N-G; TEM images of (**d**) G and (**e**) N-G; AFM images of (**f**) G and (**g**) N-G.

Figure 2a shows a representative SEM image of the graphite before liquid-phase exfoliation or ball milling, indicating an average particle size of around 30 μm. In comparison, Figure 2b,c shows representative SEM images of the agglomerations of G and N-G that exhibit a significant particle-size reduction down to 1~5 μm. The agglomeration could be a consequence of SEM sample preparation. In addition, TEM images (Figure 2d,e) show that both G and N-G present few-layered two-dimensional nanosheet-like structures with lateral sizes ranging from 400–600 nm. By analyzing the line scan profiles of AFM images (Figure 2f,g) from A to B and C to D respectively, it was possible to obtain the thickness of G and N-G (1 to 3 nm), which is similar to few-layered graphene nanosheets. These results indicate that the fabricated G and N-G have similar surface morphologies and nanostructures.

**Figure 3:**
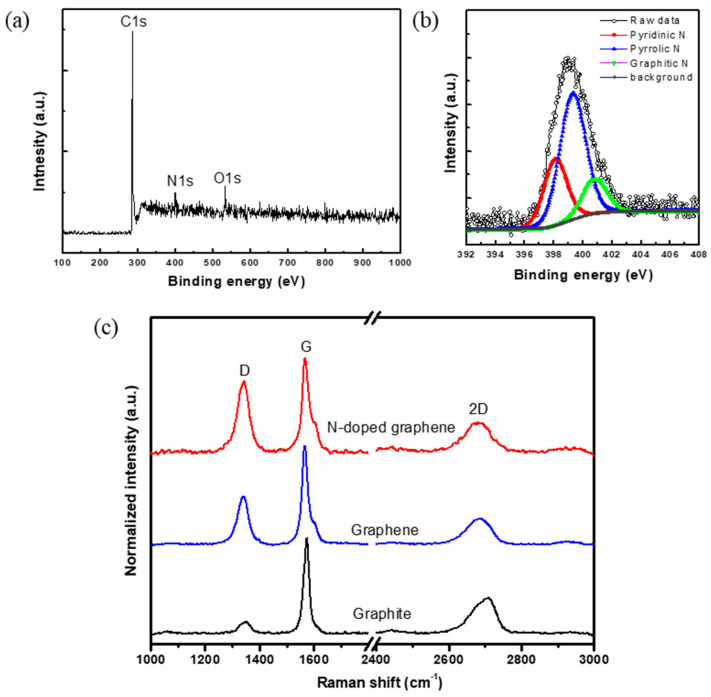
(**a**) XPS and (**b**) N1s HRXPS spectra of N-G nanosheets. (**c**) Raman spectra of graphite, G and N-G.

Figure 3a shows the XPS scan spectra, exhibiting C1s and O1s peaks at 284.6 and 534.0 eV. The N1s peak at 398.9 eV demonstrates the presence of the N atoms in the structure of N-G. Moreover, XPS analysis of N-G clearly shows the presence of N, C, and O atoms with an atomic content of 92.7%, 3.1%, and 4.2%, respectively, indicating that the fabricated samples are metal-free nanomaterials. XPS (HRXPS) results (Figure 3b) provide additional evidence of the incorporation of N atoms into the G lattice. The N1s HRXPS spectrum describes the N-G with three different nitrogen doping configurations, including pyridinic N centered at 398.2 eV (26.17 at.%), pyrrolic N centered at 400.5 eV (55.55 at.%), and graphitic N centered at 401.3 eV (18.28 at.%). These results suggest that the N dopants have been successfully doped into the sp^2^ carbon network of G. The three N doping configurations in G can change the surface polarities of the G surface, making it more polar and hydrophilic. Moreover, the N dopants within the G lattice can further alter the band structures of the G, potentially leading to improved electrical conductivity. 

Figure 3c shows the Raman spectra of G and N-G, which were normalized with respect to the G-band at 1580 cm^−1^. The spectra also exhibit D-band and 2D band at 1335 cm^−1^ and 2690 cm^−1^ respectively. It is known that the G-band arises from the bond stretching of all sp^2^ bonded pairs while the D-band is associated with the sp^3^ defect sites [51]. The relative ratio of D-band intensity to G-band intensity (I_D_/I_G_ ratio) is associated with disorders and defects in graphene-like structures [52]. Results showed that the I_D_/I_G_ ratios of N-G are larger than G and raw graphite, suggesting that the electronic structure of the sp^2^ carbons can be changed by N doping, which could influence the electronic properties [53,54]. In addition, it also suggests that doping of N atoms into G structures may generate more electroactive sites in produced 3D bone tissue engineering scaffolds.

Table 1 summarizes the averaged sheet resistance of G and N-G. Results indicate that the sheet resistance of N-G is about 13 times lower than G, suggesting that the electrical conductivity of G can be significantly enhanced by N atom doping. 

### 3.2. Scaffold Non-Biological Characterisation

**Figure 4:**
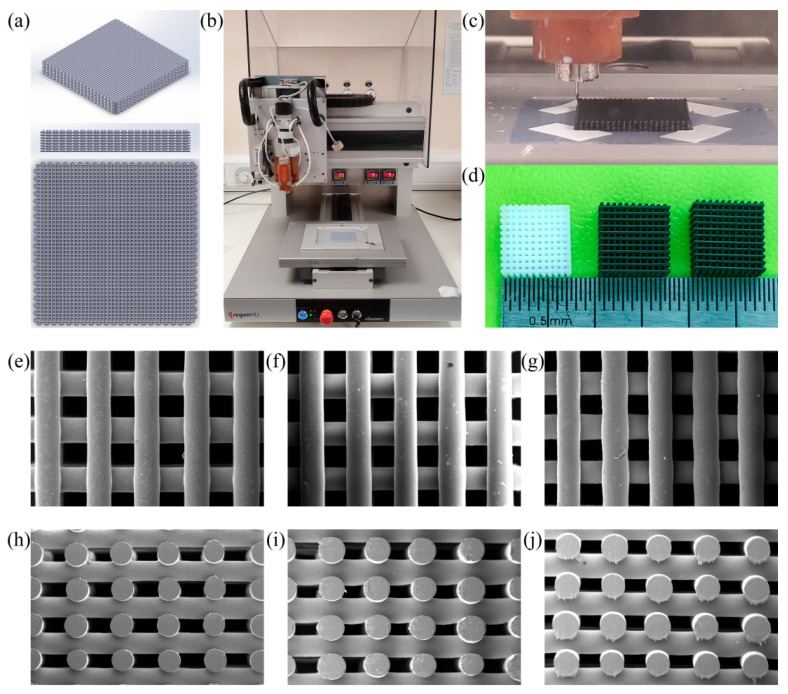
Scaffold design, fabrication, and morphological characterization. (**a**) 3D modelling (32 mm × 32 mm × 3.2 mm, 0/90° laydown pattern, 330 μm filament diameter, 680 μm filament distance); (**b**) 3D Discovery material extrusion 3D printer; (**c**) printing process; (**d**) fabricated scaffolds (cut to 11 mm × 11 mm × 3.2 mm) (left to right: PCL, PCL/G 5 wt.%, PCL/N-G 5 wt.%); SEM images of top view of scaffolds (**e**) PCL, (**f**) PCL/G 5 wt.%, and (**g**) PCL/N-G 5 wt.%; SEM images of cross-section view of scaffolds (**h**) PCL, (**i**) PCL/G 5 wt.%, and (**j**) PCL/N-G 5 wt.%.

Morphological analysis (Figure 4) compares the printed scaffolds with the designed one. Measurement results suggest the scaffold presents a regular interconnected porous structure, with filament diameter close to the nozzle diameter, ranging from 330–350 μm, confirming that this additive manufacturing technology is a viable technique to fabricate PCL, PCL/G, and PCL/N-G scaffolds with good reproducibility. Results also show that the addition of small concentrations of carbon nanomaterials has a minor influence on the scaffold morphology (around 330 μm for PCL scaffolds, 330–340 μm for PCL/G scaffolds, and 335–350 μm PCL/N-G scaffolds). All scaffolds were produced using the same combination of optimal processing parameters previously mentioned.

**Figure 5:**
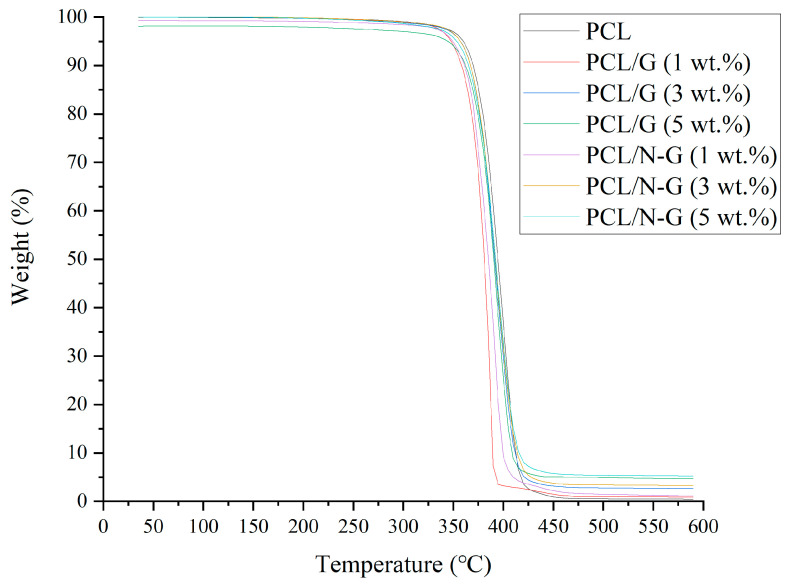
TGA characterization curves.

Figure 5 shows the TGA curves of all samples. The onset temperature of large weight loss of PCL scaffolds is 365 °C, suggesting that the PCL started to be decomposed from 365 °C, which agrees with a previous work [55]. In contrast, with the addition of 1, 3, and 5 wt.% of G and N-G into the PCL polymeric matrix, the onset decomposition temperatures were slightly decreased, ranging between 355–365 °C. This small decrease of the onset decomposition temperature of PCL/G and PCL/N-G scaffolds is due to the defects generated during the preparation of G and N-G [56]. Nevertheless, the TGA results suggest the thermal stability of PCL was almost preserved after adding G and N-G during the scaffold fabrication. Considering the chemical structures of PCL, G, and N-G, it is possible that the interaction between the PCL hydrocarbon chains and basal planes of G and N-G, and dipole–dipole attractions between the carbonate groups of PCL and oxygen-containing functional groups of G and N dopants and oxygen-containing functional groups of N-G could occur within the fabricated scaffolds. It is envisaged that those molecular interactions between PCL, G, and N-G can help to improve the arrangement of G and N-G in the PCL polymeric matrix, maintaining the thermal stability of PCL [57]. Furthermore, TGA results presented in Table 2 showed no significant carbon nanomaterial weight loss during the melt-blending or printing process, indicating that the melt-blending is a viable method to incorporate nanomaterials into the polymer matrix, without any involvement of solvent. Additionally, as all scaffolds were printed at 90 °C, processing conditions do not induce any degradation of PCL. 

Table 3 shows the WCA results at different time points (0, 15, and 30 s), which suggest that the addition of small concentration of G and N-G have a minor impact on the scaffold contact angle. However, an exception was found for 5 wt.% PCL/N-G scaffolds that presented a statistically higher WCA value than PCL scaffolds, which may be attributed to the agglomeration of carbon nanomaterials at higher concentrations, and as a consequence the carbon nanomaterial at the surface of the scaffold’s fibers may exhibit a 3D block form rather than a 2D nanosheet form. For the other groups, all PCL/G and PC/N-G scaffolds presented lower WCA than PCL scaffolds. However, only 1 wt.% PCL/G scaffolds showed a statistically significant difference. Previous studies proved that cell adhesion, modulated by protein adhesion, is strongly related to surface wettability [58]. Previous results also suggest that, depending on the cell type, moderate hydrophilic (contact angle around 30–70°) surfaces are most suitable for cell attachment and proliferation [58,59,60], while superhydrophilic (0°) and superhydrophobic (above 150°) surfaces can lead to a dramatic reduction of cell adhesion [58,59,60]. Moreover, results also seem to indicate that surface hydrophilicity and nanotopography have a major influence on mesenchymal stem cells osteoblastic differentiation and osteoblast maturation [61]. Hydrophilic surfaces have been shown to enhance osteoblast maturation [62], production of local factors [62,63], and mineralization [64] compared to hydrophobic surfaces. These observations seem to denote that the 1 wt.% and 3 wt.% carbon nanomaterial scaffolds may present more promising surface properties for cell seeding, proliferation, and differentiation.

### 3.3. Biological Characterization

**Figure 6:**
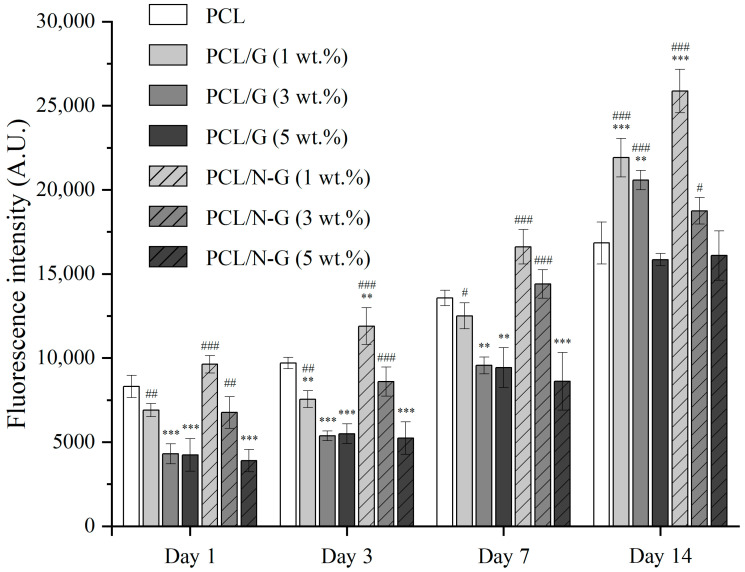
Cell viability/proliferation behavior on different scaffolds at 1, 3, 7, and 14 days after cell seeding. The significance levels were set at ** *p* < 0.01, and *** *p* < 0.001 compared with control (PCL), # *p* < 0.05, ## *p* < 0.01, and ### *p* < 0.001 compared among all graphene-loaded (G and N-G) scaffold.

Figure 6 shows the fluorescence intensity measurement results that represent the metabolic activity of cells. From day 1–14, all scaffolds showed an increasing trend in terms of fluorescence intensity, indicating no significant cytotoxicity. These results suggest that all considered scaffolds are suitable to support cell attachment and proliferation. 

At day 1 after cell seeding, PCL, 1 wt.% PCL/G, 1 and 3 wt.% PCL/N-G scaffolds showed significantly higher fluorescence intensity values than the other scaffolds, with the highest results observed for 1 wt.% PCL/N-G scaffolds. After day 3, the effect of N-G became dominant, with 1 wt.% PCL/N-G scaffolds presenting statically higher values than all the other scaffolds. PCL/G scaffolds (1 wt.% and 3 wt.%) also show a better biological performance with the cell metabolic activity, but this effect is only evident at a later stage compared to PCL/N-G scaffolds. However, 5 wt.% PCL/G, 3 wt.% PCL/N-G, and 5wt.% PCL/N-G scaffolds seem to exhibit poorer biological performance (low fluorescence intensity values) than PCL scaffolds, suggesting that the carbon nanomaterial concentration is approaching a cytotoxic threshold level (3 wt.% for N-G and 5 wt.% for G). Overall, the 1 wt.% PCL/N-G scaffold group seems to be the most suitable substrate for cell attachment and cell spreading. Results also indicate that lower concentrations of G and N-G (below a threshold) significantly enhance the biological performance of the scaffolds, this enhancement effect being more significant with N-G than G.

## 4. Conclusions

This paper successfully proved the strong potential of applying G and N-G for the fabrication of electro-active scaffolds for bone tissue engineering applications. SEM, AFM, TEM, XPS, and micro Raman results demonstrated that the N dopants were successfully integrated with G. N-G, due to its nitrogen doping, exhibits superior electrical conductivity compare to G, which improves cell-cell signaling and cell-material interactions, and improved surface hydrophilicity. This results in significantly higher biological performance as observed with hADSCs. This enhancement effect is more significant under low concentration (1 wt.%), which also reveals the potential of using lower concentrations of N-G to replace higher concentrations of G, thus minimizing the dose-dependent cytotoxicity from G. In comparison to PCL, a biocompatible material, results also suggest a threshold value of both G and N-G after which higher concentrations start to become toxic. This cytotoxicity threshold value for N-G seems to occur at lower concentration than G, but further investigations are required. At high concentration levels, after internalization, graphene induces cytotoxic effects by decreasing mitochondrial activity while increasing intracellular reactive oxygen species (ROS), caspase-3, and lactate dehydrogenase levels [65,66]. Furthermore, PCL/G electro-active scaffolds showed significant potential to enhance in vivo angiogenesis and osteogenesis effect, ultimately leading to enhanced bone tissue engineering efficacy [28]. Therefore, we can expect to achieve significantly better results with the proposed PCL/N-G scaffolds.

## Figures and Tables

**Table 1:** Electrical conductivity measurement result.

Sample	Averaged Sheet Resistance (10^3^ Ω/sq)
G	377.01 ± 10.23
N-G	28.16 ± 1.13

**Table 2:** TGA characterization results (n = 5).

Designed Scaffold	TGA Result (wt.%)
PCL	/
PCL/G (1 wt.%)	0.963 ± 0.007
PCL/G (3 wt.%)	2.611 ± 0.002
PCL/G (5 wt.%)	4.903 ± 0.003
PCL/N-G (1 wt.%)	1.070 ± 0.005
PCL/N-G (3 wt.%)	3.279 ± 0.002
PCL/N-G (5 wt.%)	5.227 ± 0.002

**Table 3:** Water-in-air contact angle measurement results (n = 4). The significance levels were set at * *p* < 0.01 and ** *p* < 0.001 compared with control (PCL).

Time	PCL	PCL/G (1 wt.%)	PCL/G (3 wt.%)	PCL/G (5 wt.%)	PCL/N-G (1 wt.%)	PCL/N-G (3 wt.%)	PCL/N-G (5 wt.%)
0 s	88.11°± 2.00°	79.97° ± 0.98° **	84.15° ± 3.85°	86.74° ± 3.36°	84.91° ± 2.29°	88.01° ± 1.18°	95.29° ± 2.54° *
15 s	86.72°± 2.37°	78.94° ± 0.79° *	83.13° ± 3.92°	85.42° ± 3.91°	84.00° ± 2.28°	87.25° ± 0.61°	95.02° ± 2.58° *
30 s	86.41°± 2.45°	78.51° ± 0.92° *	82.33° ± 4.19°	85.05° ± 3.87°	83.66° ± 2.24°	87.00° ± 0.60°	94.87° ± 2.59° *

## Data Availability

The data presented in this study are available on request from the corresponding author.
